# Correction to: ‘Using naturalistic incubation temperatures to demonstrate how variation in the timing and continuity of heat wave exposure influences phenotype’ (2022) by Breitenbach *et al.*

**DOI:** 10.1098/rspb.2022.1212

**Published:** 2022-06-29

**Authors:** Anthony T. Breitenbach, Amanda W. Carter, Ryan T. Paitz, Rachel M. Bowden

*Proc. R. Soc. B* **287**, 20200992. (Published online 5 August 2020) (http://dx.doi.org/10.1098/rspb.2020.0992)

For figure 5:

— The legend/key for the graph (on the far right side of the figure) needs to be corrected so that ‘*Cry19A1*’ is updated to read ‘*Cyp19A1*’.

— The ‘1’ at the bottom of the *y*-axis in both panels of the figure needs to be changed to ‘0’.

**Figure 5.**
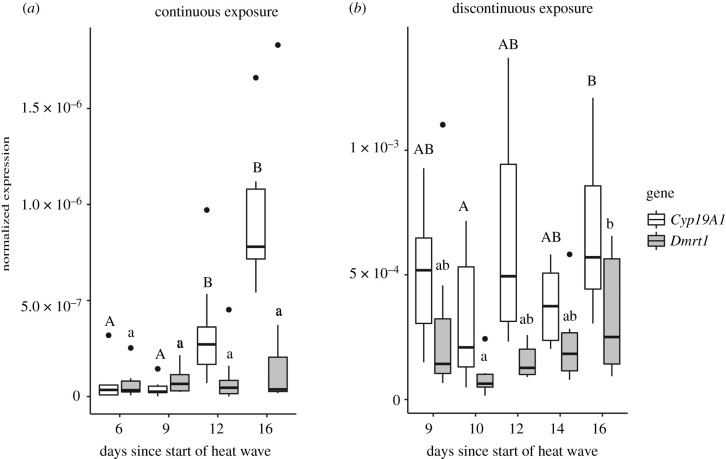
(*a*) Changes in *Cyp19A1* and *Dmrt1* expression over the course of a heat wave. The analysis was performed using both gonads from each sampled embryo. Sample sizes are listed as follows (days since start of heat wave: embryos sampled): 6: 6, 9: 6, 12: 10, 16: 7. (b) Changes in *Cyp19A1* and *Dmrt1* expression following a heat wave. The analysis was performed using both gonads from each sampled embryo. Sample sizes are listed as follows (days since start of heat wave:embryos sampled): 9: 7, 10: 8, 12: 8, 14: 8, 16: 9. The heat waves used in both conditions started on day 24 of incubation. Data shown with the median (thick black line), 25th and 75th percentiles (lower and upper boundaries of box plots, respectively), maximum and minimum values (whiskers) and outliers (closed circles). Sampling points that do not share an uppercase letter had significantly different levels of *Cyp19A1* expression, while sampling points that do not share a lowercase letter had significantly different levels of *Dmrt1* expression.

